# Evaluation of Continuous Renal Replacement Therapy and Therapeutic Plasma Exchange, in Severe Sepsis or Septic Shock in Critically Ill Children

**DOI:** 10.3390/medicina55070350

**Published:** 2019-07-07

**Authors:** Fatih Aygün, Fatih Varol, Cansu Durak, Mey Talip Petmezci, Alper Kacar, Hasan Dursun, Ahmet Irdem, Haluk Çokuğraş, Yıldız Camcıoğlu, Halit Çam

**Affiliations:** 1 Department of Pediatric Intensive Care Unit, Istanbul University Cerrahpasa Medical Faculty, Fatih, Istanbul 34098, Turkey; 2 Department of Pediatric Intensive Care Unit, Health Sciences University, Okmeydani Training and Research Hospital, Istanbul 34384, Turkey; 3 Department of Pediatrics, Health Sciences University, Okmeydani Training and Research Hospital, Istanbul 34384, Turkey; 4 Department of Infectious Disease, Istanbul University Cerrahpasa Medical Faculty, Fatih, Istanbul 34098, Turkey

**Keywords:** haemofiltration, plasma exchange, sepsis, critically ill children

## Abstract

*Background and objective:* Severe sepsis and septic shock are life-threatening organ dysfunctions and causes of death in critically ill patients. The therapeutic goal of the management of sepsis is restoring balance to the immune system and fluid balance. Continuous renal replacement therapy (CRRT) is recommended in septic patients, and it may improve outcomes in patients with severe sepsis or septic shock. Therapeutic plasma exchange (TPE) is another extracorporeal procedure that can improve organ function by decreasing inflammatory and anti-fibrinolytic mediators and correcting haemostasis by replenishing anticoagulant proteins. However, research about sepsis and CRRT and TPE in children has been insufficient and incomplete. Therefore, we investigated the reliability and efficacy of extracorporeal therapies in paediatric patients with severe sepsis or septic shock. *Materials and methods:* We performed a multicentre retrospective study using data from all patients aged <18 years who were admitted to two paediatric intensive care units. Demographic data and reason for hospitalization were recorded. In addition, vital signs, haemogram parameters, and biochemistry results were recorded at 0 h and after 24 h of CRRT. Patients were compared according to whether they underwent CRRT or TPE; mortality between the two treatment groups was also compared. *Results:* Between January 2014 and April 2019, 168 septic patients were enrolled in the present study. Of them, 47 (27.9%) patients underwent CRRT and 24 underwent TPE. In patients with severe sepsis, the requirement for CRRT was statistically associated with mortality (*p* < 0.001). In contrast, the requirement for TPE was not associated with mortality (*p* = 0.124). *Conclusion:* Our findings revealed that the requirement for CRRT in patients with severe sepsis is predictive of increased mortality. CRRT and TPE can be useful techniques in critically ill children with severe sepsis. However, our results did not show a decrease of mortality with CRRT and TPE.

## 1. Introduction

Severe sepsis and septic shock are life-threatening organ dysfunctions and causes of death in critically ill patients [1,2]. Treatment of severe sepsis and septic shock is described by the Surviving Sepsis Campaign, and it includes early recognition, microbial source control, rapid and appropriate treatment with antimicrobial agents, and goal-directed haemodynamic, ventilatory, and metabolic therapies [3]. When sepsis is not treated correctly and quickly, all organs can be affected, and each developing organ failure increases the risk of mortality. Especially, fluid balance and renal dysfunction are associated with prognosis in these patients [4]. In addition, sepsis is characterized by a dysregulated immune response to infections that involves complex interactions between endothelial cells, platelets, leucocytes, the coagulation system, and multiple inflammatory mediators [5]. Therefore, the therapeutic goal of the management of sepsis is restoring balance to the immune system and fluid balance [3].

Continuous renal replacement therapy (CRRT) is an extracorporeal technique and is recommended in septic patients who develop acute kidney injury (AKI) [6]. Continuous venovenous haemofiltration (CVVH) is a CRRT sub-type that may improve outcomes in patients with severe sepsis or septic shock [7]. Therefore, CVVH is widely used to manage to AKI and cytokine removal in severe sepsis or septic shock [7,8].

Therapeutic plasma exchange (TPE) is another extracorporeal procedure that involves separating the plasma from whole blood and removing large molecular weight substances from the plasma. After this separation, plasma is discarded and replaced with either fresh frozen plasma or albumin [9]. TPE can improve organ function by decreasing inflammatory and anti-fibrinolytic mediators and correcting haemostasis by replenishing anticoagulant proteins and factors [10]. In the literature, TPE in children improved the 28-day survival in severe sepsis with thrombocytopenia-associated multi-organ failure (TAMOF) [11,12]. However, sepsis and TPE in children have not been adequately studied. In addition, no paediatric studies have simultaneously evaluated the extracorporeal techniques, CRRT and TPE, in severe sepsis.

In this study, we investigated the reliability and efficacy of extracorporeal therapies in paediatric patients with severe sepsis or septic shock.

## 2. Materials and Methods

### 2.1. Study Design

We performed a multicentre retrospective study. Healthcare provision for children aged <18 years is provided in two paediatric intensive care units (PICUs). The first centre is equipped with seven beds, seven ventilators, two isolation rooms, and two Prismaflex^®^ haemofiltration machines (Baxter, Deerfield, IL, USA). The second centre is equipped with 12 beds, 11 ventilators, two isolation rooms, and two Prismaflex^®^ haemofiltration machines. The data of all patients admitted to the PICUs for various critical illnesses between January 2014 and April 2019 were extracted from electronic and written medical records (in accordance with the ethical principles for medical research), and patients with severe sepsis (or septic shock) on admission were included in this study.

Patients with a history of a duration of PICU stay <24 h and those who died on the first day of admission were excluded. Ethical approval with waiver of consent was obtained from each institution that approved the study (İstanbul University-Cerrahpaşa, ethical committee no. 29430533-903.99-92611, December 2018, and Okmeydani Research and Training Hospital, ethical committee no. 2017-777, December 2017). All clinical investigations were conducted according to the principles expressed in the Declaration of Helsinki of 1975, revised in 2013 [13].

We recorded all materials, data, computer codes, and protocols associated with the publication for readers.

### 2.2. Patient Population and Data Collection

Demographic data and reason for hospitalization were recorded. The patients’ sex, age, invasive or non-invasive mechanical ventilation requirement, duration of hospitalisation in the PICU, and mortality were recorded. In addition, vital signs, haemogram parameters, and biochemistry results were recorded at 0 h and 24 h of CRRT. The patients were compared according to if they underwent CRRT or TPE; mortality between the two treatment groups was also compared.

Patients with the diagnosis of sepsis at admission to intensive care unit were included in the study. Patients with sepsis/septic shock were enrolled in the study using criteria according to the Surviving Sepsis Campaign guidelines [3]. Severe sepsis was defined as the presence of at least two criteria out of four SIRS criteria, plus suspected infection with at least one organ dysfunction.

The PRISM III score was calculated via a tool available on the Turkish Ministry of Health website. This tool calculates the PRISM score based on the values for the lowest/highest systolic blood pressures from the first 24 h, diastolic blood pressure, heart rate, respiratory rate, PaO_2_/FiO_2_, PaCO_2_, prothrombin time/partial thromboplastin time, serum total bilirubin, calcium, potassium, glucose, bicarbonate, pupillary response, and Glasgow coma score.

We defined AKI as oliguria (urine output of <0.5 mL/kg/h for 6 h) and an elevated serum creatinine value for the patient’s age or a 1.5-fold increase in the creatinine concentration at 24 h (relative to admission). The estimated glomerular filtration rate was calculated according to the original Schwartz formula, based on serum urea and creatinine concentrations that were measured using standard laboratory procedures.

### 2.3. Catheterisation

CRRT indications included fluid overload, electrolyte imbalance, and metabolic acidosis in our sepsis patients. Some patients had more than one indication. Double-lumen central venous catheters were percutaneously placed along the femoral vein, jugular vein, or subclavian vein. Primarily, jugular and femoral catheters were inserted due to the potential complications. The location of all jugular and subclavian catheters was controlled by chest X-ray. The right side of the patient was favoured for the insertion of the jugular and subclavian catheters due to decreased complication risk.

All patients received sedation and analgesia prior to catheterization. Midazolam, ketamine, and morphine were preferred for sedation and analgesia. Lidocaine was used as a local anaesthetic. Neuromuscular blockers were not used. Surgical catheters were not attached (cut-down, etc.). The aseptic method was used during catheterisation. The entry location was sterilised using 1% povidone-iodine and allowed to dry. Disposable sterile covers that covered the whole body were used. Sterile aprons, masks, and bonnets were used. The catheters were attached using the Seldinger technique and fixed to the skin with silk sutures.

### 2.4. CRRT Protocol

CRRT was performed in CVVH using the Prismaflex^®^ (Baxter, USA) haemofiltration system and poly membrane (AN69) filters. Only three patients used oXiris^®^ adsorbing membranes (Baxter, USA). The blood flow rate was 5–20 mL/kg/min. The dialysate rate, replacement fluid rate, and ultrafiltration rate were adjusted on the basis of the patients’ diagnoses. The dialysate flow rates were set to 2000 mL/1.73 m^2^/h. Prismocitrate^®^ (18.0, Baxter, USA), Dialisan^®^ (Baxter, USA), PrismOcal^®^ (Baxter, USA), and Multibic^®^ (Fresenius Medical Care, AG Co., Homburg, Germany) were used as dialysate solutions.

Anticoagulation was performed using unfractionated heparin and citrate. Heparin was used at a dosage of 5–25 UI/kg/h. The dosage of heparin was adjusted on the basis of the activated partial thromboplastin time which was checked every 4 h and maintained at 60–80 s. Blood gas analysis was used to check Ca++ arterial, Ca++ pre-pump, Ca/Ca++, lactate, and bicarbonate (HCO_3_) for citrate anticoagulation-use patients.

We used the CRRT for the patients having progressive metabolic acidosis, volume overload or oliguria, and severe electrolyte abnormalities. Although the physicians of both intensive care units were trained in the same center and followed the same procedures, we did not have a written protocol for consensus in our study. As a general rule, CRRT was initiated in the patients having pH < 7.1, HCO_3_ < 10, lactate > 4 mmol/L, volume overload of more than 10%, oliguria, or electrolyte disturbance despite supportive treatments.

### 2.5. TPE Protocol

We performed plasma exchange using a filter membrane-based apparatus. TPE was performed using the Prismaflex^®^ (Baxter, USA) system, and TPE 1000 or TPE 2000 sets were used. The amount of plasma was calculated as follows: estimated plasma volume (L) = 0.07 × weight (kg) × (1 − haematocrit). We used fresh frozen plasma for the replacement fluid. Saline 0.9% was used to prime the TPE circuit. A heparin bolus was administered at the start, and 5–25 U/kg was administered hourly. During the TPE procedures, blood flow was adjusted to 50–150 mL/min. Vital signs were thoroughly monitored during TPE procedures. Control blood samples were taken immediately before and after TPE. Antibiotic, IVIG, hydrocortisone, antiepileptic, and similar drugs were given after TPE sessions. Therefore, the drugs were not cleared by TPE.

Despite standard treatment according to the survival sepsis campaign, TPE was performed in patients with hemodynamic instability such as tachycardia, hypotension, increasing need for inotropic drugs. TPE was also administered in patients with severe liver dysfunction, coagulation disorder and persistent lactic acidosis. However, we did not have a protocol or consensus in both of the centers. Therefore, we do not have a certain cut-off values or indications. TPE was decided to start depending on the initiative and experience of the intensive care staff.

### 2.6. Statistical Analysis

Statistical analysis was performed using SPSS software (version 21.0, IBM Corporation, Armonk, NY, USA). Numerical data were expressed as median values (min-max), while categorical data were expressed as frequency (n) and percentage (%). Comparisons of the differences in the baseline characteristics were performed using Student’s t-test for parametric data and the Mann-Whitney U-test for non-parametric data. Categorical variables were compared using a chi-square test or Fisher’s exact test. Univariate binary logistic regression models were employed to calculate the odds ratios (ORs) with 95% confidence interval (CIs) for risk factors associated with infection occurrence. Survival data were calculated using Kaplan–Meier log-rank statistical analysis. A value of *p* < 0.05 was considered statistically significant.

## 3. Results

### 3.1. Cohort Flow

**Figure 1:**
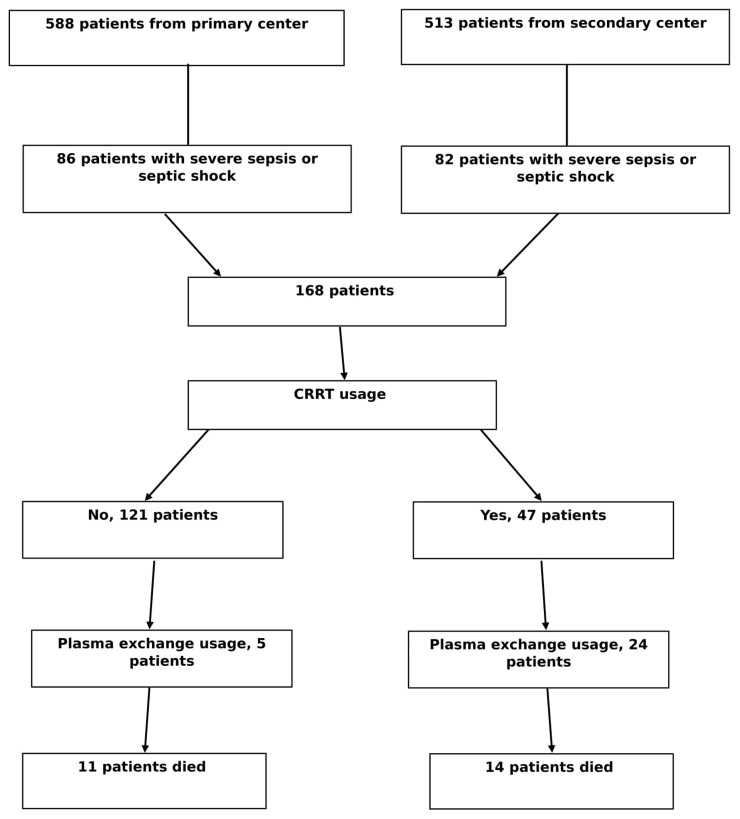
Cohort flow, CRRT, continuous renal replacement therapy.

Between January 2014 and April 2019, 1101 children were admitted to the PICUs; 588 patients from the primary centre and 513 patients from the secondary centre. Of them, 168 were eligible for the present study. Forty-seven (27.9%) patients with sepsis underwent CRRT. In addition, 24 patients underwent TPE, and 14 patients died (Figure 1).

### 3.2. The Relationship between Severe Sepsis and CRRT Regarding Prognostic Factors in the PICU

There was a statistically significant relationship between severe sepsis with CRRT usage and age, inotropic drug use, albumin use, red blood cell (RBC) transfusion, mortality, blood calcium, alanine transaminase (ALT), aspartate aminotransferase (AST), PRISM III score, lactate dehydrogenase (LDH), and platelet count (Table 1).

### 3.3. The Relationship between Severe Sepsis and TPE Regarding Prognostic Factors in the PICU

There was a statistically significant relationship between severe sepsis with TPE and inotropic drug use, albumin use, RBC transfusion, mortality, blood calcium, ALT, AST, LDH, uric acid, haemoglobin, and platelet count (Table 2).

### 3.4. Comparison of Prognostic Factors for Mortality in Patients with Severe Sepsis

There was a statistically significant relationship between mortality and invasive mechanical ventilation (IMV) support, inotropic drug use, AKI, CRRT, PRISM III score, the duration of stay in the PICU, and 0 h lactate level in patients with severe sepsis (Table 3).

### 3.5. Logistic Regression Analysis of Risk Factors for Mortality in Patients with Sepsis

According to logistic regression analysis, the ORs were 3.996 (95% CI, 1.126–14.186) for CRRT, 7.287 (95% CI, 1.666–31.887) for IMV use, and 10.638 (95% CI, 1.180–95.906) for inotropic drug use (Table 4).

### 3.6. Changes in Serum Liver and Blood Gases in Patients with Severe Sepsis with or without Continuous Haemofiltration

The patients were divided into three groups as CRRT, CRRT-TPE, and symptomatic treatment. When the demographic and prognostic findings of the patients were compared, the age of the patients treated with CRRT + TPE was significantly higher. In addition, RBC, mortality, and multi-organ failure were significantly lower in the symptomatic group. There were no significant differences between the groups for underlying disease at admission. There was no difference between CRRT and CRRT + TPE patients in terms of initial settings, starting time, catheters’ diameters, and duration. Haemodialysis filters with larger surface area were used in CRRT + TPE group.

Biochemistry results and blood gases were recorded at the start of (0 h) and after 24 h of CRRT. In our study, ALT, AST, and lactate values at 0 h and 24 h were significantly lower in the symptomatic treatment group. The most significant decrease in 0–24 h ALT levels was in the group that underwent CRRT and TPE simultaneously. The most significant decrease in 0–24 h AST was in the group that underwent CRRT. No changes were found in HCO_3_ levels between the 0 h and 24 h groups (Table 5).

### 3.7. Survival Data from Kaplan–Meier Survival Analysis

**Figure 2:**
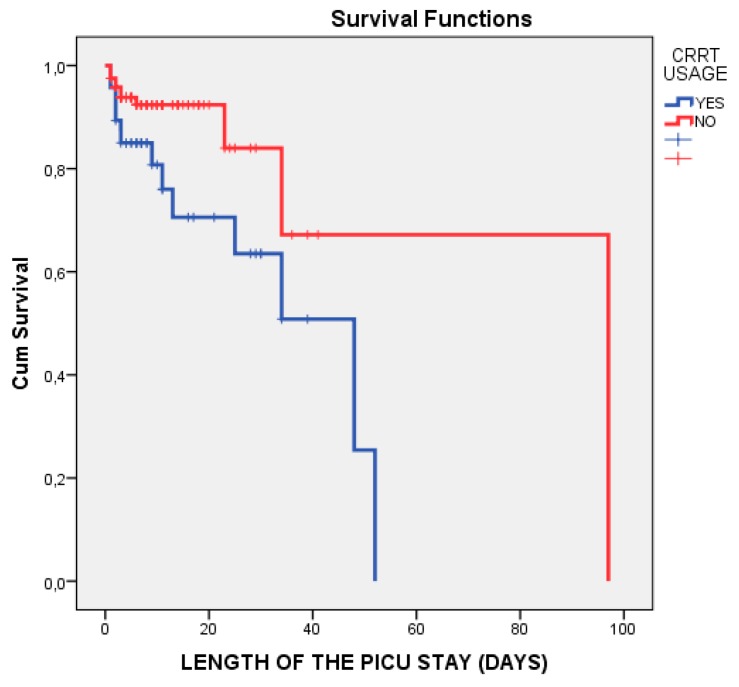
Kaplan–Meier log-rank statistic for testing the survival distribution between CRRT requirement and mortality during the PICU stay. CRRT, continuous renal replacement therapy; PICU, paediatric intensive care unit.

Kaplan–Meier log-rank statistic for testing the survival distribution between CRRT requirement and mortality during the PICU stay demonstrated that there was a significant difference between the groups for CRRT (*p* = 0.015) (Figure 2).

**Figure 3:**
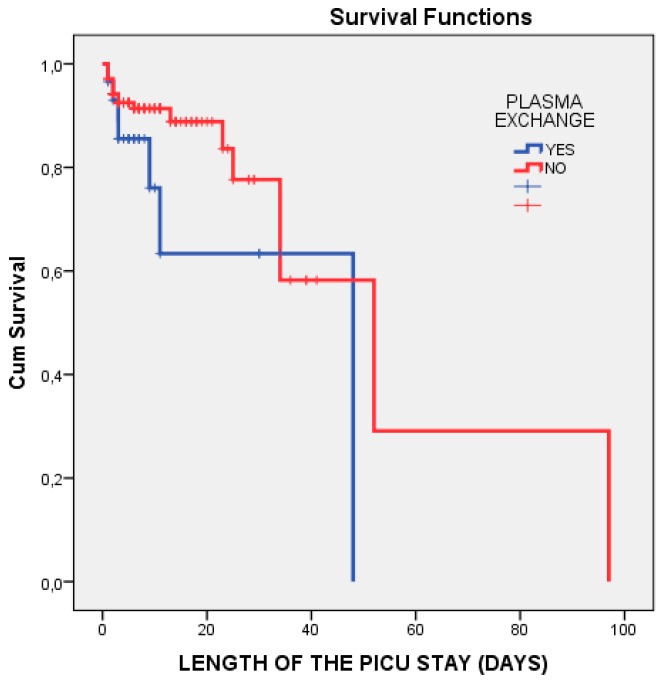
Kaplan–Meier log-rank statistic for testing the survival distribution between TPE requirement and mortality during the PICU stay. TPE, therapeutic plasma exchange; PICU, paediatric intensive care unit.

The Kaplan–Meier log-rank statistic for testing the survival distribution between TPE requirement and mortality during the PICU stay demonstrated that there was no significant difference between the groups (*p* = 0.110) (Figure 3).

## 4. Discussion

The present multicentre study revealed that CRRT requirement in patients with severe sepsis was associated with poor outcomes in critically ill children. However, there was no relationship between TPE requirement and prognosis. Among the 168 enrolled patients with sepsis, 47 (27.9%) underwent CRRT and 24 underwent TPE. In patients with severe sepsis, the need for IMV, CRRT, and inotropic drugs was statistically associated with mortality. There was no correlation between AKI and mortality in the logistic regression analysis. However, there was a significant relationship between CRRT need and mortality. In this retrospective study, the use of CRRT and TPE have no positive effect for sepsis patients’ prognosis.

The Surviving Sepsis Campaign guidelines suggest using CRRT to recover fluid balance in haemodynamically unstable patients with sepsis or septic shock [3]. The majority of our patients were haemodynamically unstable. In addition, the median age of the patients who underwent CRRT in our study was 2.0 years. Small body weight poses a problem for invasive procedures and intermittent dialysis; therefore, we avoided the use of intermittent haemodialysis for AKI in our patients.

High-volume CVVH is an accepted treatment modality for critically ill patients with severe sepsis [8]. It improves acid-base and fluid homeostasis, modulates the immune system response, and comprehensively reduces the concentration peaks of a variety of inflammatory mediators. Thus, CVVH can correct organ injury and dysfunction [6,7,8,14]. We followed a slightly different method than that in the literature. We used CVVHDF (continuous venovenous hemodiafiltration) with high volume haemofiltration and a standard dialysate rate in some patients with sepsis. We preferred haemofiltration not only for AKI but also for reducing cytokine storms. The most important goal of adding dialysate was to regulate the acid-base and electrolyte balance. Especially, we have kept dialysate rates higher than standard CRRT usage in the patients with refractor lactate elevation. We slowly decreased the dialysis rate to avoid rebound of lactate elevation, when lactate and metabolic acidosis were reduced. Another important result in our study was the relationship between lactate level and mortality. Lactate levels were significantly higher in patients who died. However, in our study, no positive effect of CRRT or CRRT + TPE on lactate clearance was shown.

In the literature, patients with severe sepsis or septic shock exhibit metabolic acidosis at intensive care unit admission [15]. High volume haemofiltration can effectively correct severe lactic acidosis [16]. Furthermore, it can also improve inflammation-damaged organ function, particularly hepato-renal function, and greatly improve oxygenation in patients with acute respiratory failure [17]. In our study, CRRT and TPE were used considering the same indications as in the literature, such as hepato-renal function disorders and metabolic acidosis. The rate of lactate elevation, low HCO_3_, showed metabolic acidosis was related to CRRT. In addition, significant elevation of ALT and AST values in both CRRT and TPE patients suggest liver damage. Interestingly there was no association with the international normalised ratio (INR) in our study. These findings showed that liver synthesis functions were normal which suggested possible necrosis, such as cardiac or circulatory failure.

In a recent study, it was shown that continuous haemofiltration significantly improves mortality in paediatric patients with bacterial sepsis-associated liver dysfunction [18]. In contrast to that study, we did not find a significant improvement in ALT levels with CRRT alone. However, a correlation was found between the 0-h and 24-h liver enzymes in CRRT-TPE use. As shown in Table 5, a significant decrease in ALT values was achieved with CRRT-TPE use at 24 h. A similar association was demonstrated for AST. In both CRRT and CRRT-TPE, the AST values were significantly improved compared to the symptomatic group.

Some studies have suggested that TPE use in liver failure decreases the need for vasopressors, induces regression of hepatic encephalopathy, and positively affects body nitrogen balance [19]. However, in their 2016 guidelines, the American Society for Apheresis only weakly recommended TPE for patients with sepsis [10] because there were not enough evidence-based studies for TPE use in sepsis. In our study, CRRT-TPE was used in patients with higher liver enzymes and lactate levels; an improvement in liver function was observed in this group. However, there was no significant decrease between survival and exitus patients’ liver enzymes as shown in Table 3.

Another important aspect of our study was the increase in the requirement for CRRT and TPE in patients with LDH elevation and lower platelet count, which may have been due to TAMOF. Şevketoğlu et al. proposed early plasma exchange for the recovery of ADAMTS13 (a disintegrin and metalloproteinase with thrombospondin-1 repeats, member 13) activity and the coagulation system in TAMOF patients [11]. We performed TPE in patients with multi-organ failure, severe coagulation, and liver dysfunction. Therefore, the number of patients with TAMOF was significantly higher among patients who underwent TPE. ADAMTS13 was not measured in our patients. In addition, no significant correlation was found between coagulation values and CRRT or TPE. The main relationship was determined by liver dysfunction, thrombocytopenia, and LDH. Thrombocytopenia is common in patients receiving CRRT [20]. A low platelet count at admission was a risk factor for CRRT in our study. The threshold for platelet transfusion was 20 10^3^/µL in stable patients and 50 10^3^/µL in CRRT patients with heparin use. In our study, there was only one patient with hemolytic uremic syndrome and septic shock. This patient was treated with CRRT and supportive treatment, similar to TAMOF.

In our study, CRRT was associated with mortality in patients with severe sepsis. However, the same relationship was not observed for TPE. The mortality rate was approximately four times higher in patients with a requirement for CRRT than in patients without needed CRRT. This relationship is actually a result because our study is retrospective. As it is seen in Table 1, the higher PRISM III score, inotrope requirement and organ failure were seen in the first day of CRRT. In addition, as shown in Table 5, lactate levels were significantly higher in patients undergoing CRRT or CRRT + TPE. In fact, CRRT was applied in the children who were most severe clinical conditions in our study. A similar relationship has also been reported in the literature. A recent study demonstrated that early mortality following CRRT initiation was high in critically ill patients undergoing CRRT [21]. In addition, mortality was significantly higher in patients with sepsis who needed inotropic drugs and IMV. The most important factor associated with mortality was the requirement for inotropic drugs.

In the literature, some studies have indicated that CRRT can improve oxygenation in patients with AKI and respiratory failure [22,23]. Respiratory function is frequently impaired because of pulmonary congestion and increased vascular permeability in patients with AKI [24]. However, in our study, there was no significant correlation between the use of CRRT (or TPE) and the need for IMV.

In a retrospective multicentre study, Lima et al. showed that the need for TPE was associated with mortality in PICUs [25]. In this study, 887 patients underwent TPE, and 45% had multi-organ failure. The mortality rate of TPE patients was higher than that of patients without TPE. This result could be related to comorbidities, AKI, or multi-organ failure in the patients. In contrast, TPE use did not increase the mortality rate in our study; moreover, there was no significant relationship between TPE and multi-organ failure. However, we revealed a positive effect on liver function with TPE-CRRT use.

A previous study reported that the need for RBC transfusion increased sepsis mortality in critically ill children [26]. Numerous mechanisms affect this situation, such as reduced oxygen release, transfusion-related immunomodulation, or the procoagulant effects of RBC-derived microparticles [27,28]. In our study, there was a significant association between mortality and RBC transfusion in patients with severe sepsis. In both centres, the RBC transfusion protocol was the same because the physicians were educated at the same University. However, our RBC transfusion rates may have been higher than expected, because we aimed to keep Hgb > 10 g/dL in patients with severe sepsis and shock (3). Our patients were very young; therefore, we had to transfuse almost all patients who underwent CRRT. In patients with improved shock symptoms and better general condition, our Hgb transfusion limit is 7 g/dL.

In the study reported by Shum et al., oXiris^®^-CVVH was delivered to six patients with septic AKI, and the mortality score was better in the oXiris^®^ group than in the other dialysate filters groups. However, no significant difference was observed in the mortality rate [29]. AN69 and a small number of oXiris^®^ filters were used for CRRT in our study. Therefore, we could not evaluate the effectiveness of oXiris^®^.

In our study, the age of patients who underwent TPE was significantly higher than CRRT. The youngest patient with TPE was aged 5 months and 7.8 kg and the youngest patient with CRRT was aged 2 days and 2.9 kg. Since CRRT could not be performed in our neonatal intensive care unit, this patient was transferred to PICU and CRRT was applied. The most important reason for using TPE in low weight patients was the high volume of filters. TPE 1000, the smallest filter, had a priming volume of 71 mL and required blood priming in <10 kg children. When TPE could not be performed, supportive treatment was continued.

Although they were young and had severe sepsis, no serious complications (e.g., pneumothorax or severe haemorrhage) occurred in our patients. Catheter-related haemorrhage, vascular thrombosis, and sepsis were observed; however, CRRT use or catheter insertion was not associated with complications.

In a retrospective study, Sole et al. evaluated 21 paediatric patients with refractory septic shock. In this study, it was reported that ECMO treatment, which will be started in the early period, may benefit survival [30]. In our study, ECMO support is provided at both the centres. However, our success rates in septic shock are very low and we do not have enough experiences. In the first centre, two patients underwent ECMO due to septic shock and both cases were fatal. Therefore, these patients were not included in our study. In our units, patients with myocarditis and respiratory failure have better survival ratio.

Coupled plasma filtration adsorption (CPFA) is an extracorporeal blood purification treatment in the patients with septic shock. CPFA contains two parts; firstly, it is plasma separation and adsorption of cytokines. Secondly, it is hemofiltration for remove fluid and cytokines [31]. However, it is a support treatment modality that is not known and applied too much, especially in our country. We did not use in any patient.

Our study had some limitations. First, this was a retrospective observational study with a limited number of patients. These findings need to be confirmed in a prospective study with larger sample size. Second, the numbers of TPE sessions were not recorded. Third, we could not include all the laboratory results during the extracorporeal treatments. Fourth, this study included a heterogeneous group of patients (age, disease severity, etc.) and time of onset for TPE use. Fifth, since HCO_3_ treatment was administered to patients with deep metabolic acidosis on the first day, improvements in blood gas with CRRT may have been misleading. Sixth, patient selection in the CRRT and TPE groups may have been biased because of the retrospective nature of the study. There can be biased that CRRT-TPE was performed in patients with higher lactate and liver enzymes. However, a major strength of our study is that no studies have examined simultaneous TPE and CRRT in children with severe sepsis.

## 5. Conclusions

The present multicentre study revealed that the requirement for CRRT in severe sepsis can be a predictive factor of increased mortality in critically ill children. CRRT and TPE can used in critically ill children for severe sepsis with organ failures. Although both CRRT and TPE improved liver function tests, these extracorporeal technics did not decrease the mortality. In conclusion, our results did not show a decrease of mortality in the patients with CRRT and TPE usage.

## Figures and Tables

**Table 1:** Comparison of prognostic factors in patients with sepsis with or without CRRT.

		CRRT during Sepsis		*p-*Value
		Yes (*n* = 47), Median (Min–Max)	No (*n* = 121), Median (Min–Max)	
Sex (male)		24 (51.1%)	62 (51.2%)	0.984
Age, years		2.0 (2 days–17.83 years)	1.0 (3 days–17.50 years)	0.022
IMV support		24 (51.1%)	57 (47.1%)	0.541
Inotropic drug use		34 (72.3%)	59 (48.8%)	0.006
Albumin use		37 (78.7%)	58 (47.9%)	<0.001
RBC transfusion		45 (95.7%)	86 (71.1%)	<0.001
IVIG use		22 (46.8%)	38 (31.4%)	0.133
NIV support		26 (55.3%)	59 (48.8%)	0.445
Mortality		14 (29.8%)	11 (9.1%)	<0.001
Duration of stay in PICU		7.0 (26 h–52 d)	6.0 (28 h–97 d)	0.085
PRISM III score		28 (5–56)	20 (3–65)	0.038
Multi-organ failure		29 (61.7%)	43 (35.5%)	0.006
Laboratory findings	Sodium (mmol/L)	136.0 (118.0–182.0)	138.0 (119.0–160.0)	0.750
	Chlorine (mmol/L)	99.0 (84.2–150.0)	102.4 (84.8–121.0)	0.368
	Calcium (mg/dL)	8.2 (5.6–10.6)	9.1 (6.4–11.3)	<0.001
	Magnesium (mg/dL)	1.85 (0.90–4.00)	2.00 (0.89–4.11)	0.652
	ALT (IU/L)	48 (5–4388)	20 (4–1620)	0.008
	AST (IU/L)	72 (19–8620)	34 (8–1954)	0.010
	PT (s)	16.8 (11.2–28.3)	15.6 (1.3–26.6)	0.300
	aPTT (s)	40.4 (26.2–124.0)	39.6 (18.8–77.5)	0.108
	INR	1.46 (0.90–2.40)	1.40 (0.80–2.20)	0.325
	LDH (IU/L)	514 (1–6310)	395 (126–2591)	<0.001
	CRP (ml/dL)	7.8 (0.03–405.9)	15.9 (0.1–467.0)	0.935
	Leucocyte count (mm^3^)	7500 (200–111,800)	12095 (13–50,180)	0.528
	Uric acid (mg/dL)	4.2 (0.1–17.8)	3.7 (0.9–16.2)	0.323
	Haemoglobin (g/dL)	8.7 (6.1–19.3)	10.0 (5.6–15.0)	0.058
	Platelet count (10^3^/µL)	133 (160–441)	252 (6–899)	<0.001
	HCO_3_ (mmol/L)	15.0 (9.0–29.5)	20.2 (3.5–37.0)	<0.001
	Lactate (mmol/L)	4.4 (0.5–16.0)	2.0 (0.5–29.0)	0.014
CRRT indications	Progressive metabolic acidosis	35 (74.5%)		
	Volume overload-oliguria	24 (51.1%)	-	-
	Severe electrolyte abnormalities	9 (19.1%)		

Abbreviations: CRRT, continuous renal replacement therapy; IMV, invasive mechanical ventilation; RBC, red blood cell; IVIG, intravenous immunoglobulin; NIV, non-invasive mechanical ventilation; PICU, paediatric intensive care unit; ALT, alanine transaminase; AST, aspartate aminotransferase; PT, prothrombin time; aPTT, activated partial thromboplastin time; INR, international normalised ratio; LDH, lactate dehydrogenase; CRP, C-reactive protein.

**Table 2:** Comparison of prognostic factors in patients with sepsis with or without TPE.

		TPE Use during Sepsis		*p-*Value
		Yes (*n* = 29), Median (Min–Max)	No (*n* = 139), Median (Min–Max)	
Sex (male)		13 (44.8%)	73 (52.5%)	0.451
Age, years		5.0 (5 months–17.83 years)	3.0 (2 days–17.58 years)	0.761
IMV support		14 (48.3%)	67 (48.2%)	0.942
Inotropic drug use		21 (72.4%)	72 (51.8%)	0.042
Albumin use		23 (79.3%)	72 (51.9%)	0.008
RBC transfusion		28 (96.6%)	103 (74.1%)	<0.001
IVIG use		13 (44.8%)	47 (33.8%)	0.260
NIV support		13 (44.8%)	72 (51.8%)	0.495
Mortality		7 (24.1%)	18 (12.9%)	0.124
Duration of stay in PICU		6.0 (27 h–48 d)	7.0 (26 h–97 d)	0.761
PRISM III score		28 (6–55)	21 (3–65)	0.195
Multi-organ failure		15 (51.7%)	57 (41.0%)	0.289
Laboratory findings	Sodium (mmol/L)	136.0 (119.0–182.0)	138.0 (119.0–161.0)	0.399
	Chlorine (mmol/L)	99.9 (84.2–150.0)	102.0 (84.8–121.0)	0.937
	Calcium (mg/dL)	8.0 (5.6–10.6)	9.0 (6.4–11.3)	<0.001
	Magnesium (mg/dL)	1.64 (1.10–4.11)	2.00 (0.89–4.00)	0.507
	ALT (IU/L)	43 (7–4388)	20 (4–1620)	<0.001
	AST (IU/L)	51 (19–8620)	36 (8–1954)	<0.001
	PT (s)	16.8 (11.2–28.3)	15.6 (1.3–26.6)	0.318
	aPTT (s)	41.6 (27.7–124.0)	39.1 (18.8–124.0)	0.226
	INR	1.42 (0.90–2.40)	1.40 (0.80–2.20)	0.455
	LDH (IU/L)	539 (1–6310)	393 (126–2591)	<0.001
	CRP (mL/dL)	8.1 (0.74–405.9)	11.6 (0.3–467.0)	0.408
	Leucocyte count (mm^3^)	7100 (200–54,600)	12095 (13–111,800)	0.216
	Uric acid (mg/dL)	5.7 (1.0–17.8)	3.5 (1.0–16.2)	0.004
	Haemoglobin (g/dL)	8.0 (6.1–11.2)	9.6 (5.6–19.3)	0.007
	Platelet count (10^3^/µL)	84 (24–410)	228 (6–899)	<0.001
	HCO_3_ (mmol/L)	13.9 (9.0–35.3)	19.2 (3.5–37.0)	0.150
	Lactate (mmol/L)	4.5 (0.5–16.0)	2.5 (0.5–29.0)	0.160

Abbreviations: IMV, invasive mechanical ventilation; RBC, red blood cell; IVIG, intravenous immunoglobulin; NIV, non-invasive mechanical ventilation; PICU, paediatric intensive care unit; ALT, alanine transaminase; AST, aspartate aminotransferase; PT, prothrombin time; aPTT, activated partial thromboplastin time; INR, international normalised ratio; LDH, lactate dehydrogenase; CRP, C-reactive protein.

**Table 3:** Comparison of prognostic factors for mortality in patients with severe sepsis.

	Mortality		*p*-Value
	Yes (*n* = 25), Median (Min–Max)	No (*n* = 143), Median (Min–Max)	
Sex (male)	12 (48.0%)	74 (51.7%)	0.729
Age, years	1.0 (2 months–17.58 years)	1.1 (2 days–17.83 years)	0.799
Invasive mechanical ventilation support	22 (88.0%)	64 (44.8%)	<0.001
Inotropic drug use	24 (96.0%)	69 (48.3%)	<0.001
Albumin use	17 (68.0%)	78 (54.5%)	0.211
Red blood cell transfusion	22 (88.8%)	109 (76.2%)	0.190
Intravenous immunoglobulin use	10 (40.0%)	50 (35.0%)	0.628
Acute kidney injury	22 (88.0%)	65 (45.5%)	<0.001
Continuous renal replacement therapy	14 (56.0%)	33 (23.1%)	<0.001
Therapeutic plasma exchange	7 (28.0%)	22 (15.4%)	0.124
PRISM III score	31 (12–65)	20.5 (3–55)	<0.001
Duration of stay in PICU	3.0 (26 h–97 d)	7.0 (27 h–41 d)	0.029
HCO_3_ (mmol/L)	16.9 (9.0–37.0)	19.1 (6.3–35.3)	0.254
Lactate (mmol/L)	4.4 (0.6–17.0)	2.4 (0.5–11.0)	0.002
ALT (IU/L)	11.0 (−234–134)	11.0 (−1762–45)	0.716
AST (IU/L)	2.0 (−640–234)	1.0 (−4523–234)	0.698

Abbreviation: PICU, paediatric intensive care unit.

**Table 4:** Logistic regression analysis of risk factors for mortality in patients with sepsis.

Risk factors	*p*-Value	Odds Ratio	95% Confidence Interval
Acute kidney injury	0.155	1.952	0.664–13.131
Continuous renal replacement therapy	0.032	3.996	1.126–14.186
Invasive mechanical ventilation	0.008	7.287	1.666–31.887
Therapeutic plasma exchange	0.738	0.797	0.211–3.008
Inotropic drug use	0.005	10.638	1.180–95.906
Blood component transfusions	0.750	1.325	0.234–7.490
Intravenous immunoglobulin	0.338	0.557	0.168–1.842
Albumin use	0.189	0.376	0.088–1.619

**Table 5:** Changes in demographic, prognostic, and laboratory results in patients with severe sepsis with or without continuous hemofiltration.

	CRRT (*n* = 23) Median (Min–Max)	CRRT+TPE (*n* = 24) Median (Min–Max)		Without CRRT or TPE (*n* = 116) Median (Min–Max)	*p-*Value
**Underlying disease before the sepsis on admission**					
Healthy before the admission	9 (39.1%)	10 (41.7%)		56 (48.3%)	0.065
Metabolic diseases	8 (34.8%)	2 (8.3%)		13 (11.2%)	
Neurologic	1 (4.3%)	3 (12.5%)		16 (13.8%)	
Haematology-oncology	2 (8.7%)	2 (8.3%)		6 (5.2%)	
Congenital heart diseases	1 (4.3%)	0		8 (6.9%)	
Others	2 (8.7%)	7 (29.2%)		17 (14.7%)	
Sex (male)	11 (47.8%)	11 (45.8%)		63 (54.3%)	0.679
Age, years	1.0 (2 days–17.58 years)	7.0 (5 months–17.83 years)		1.0 (2 days–17.50 years)	<0.001
**Prognostic factors**					
IMV support	14 (60.9%)	10 (41.7%)		57 (49.1%)	0.124
Inotropic drug use	17 (73.9%)	17 (70.8%)		58 (50.0%)	0.107
RBC transfusion	22 (95.7%)	23 (95.8%)		83 (71.6%)	0.003
IVIG use	11 (47.8%)	11 (45.8%)		38 (32.8%)	0.239
NIV support	15 (65.2%)	11 (45.8%)		58 (50.0%)	0.254
PRISM-III score	24 (5–56)	29 (6–55)		20 (3–65)	0.117
Duration of stay in PICU	9.0 (48 h–52 d)	6.0 (26 h–48 d)		7.0 (28 h–97 d)	0.153
Multi-organ failure	17 (73.9%)	12 (50.0%)		43 (37.1%)	0.017
Mortality	7 (30.4%)	7 (29.2%)		11 (9.5%)	0.026
**Catheters’ diameters**					
6.5 and 7 Fr		8 (34.8%)	1 (4.2%)	-	0.065
8 Fr		8 (34.8%)	11 (45.8%)		
10 Fr		1 (4.3%)	2 (8.3%)		
11.5 and 12 Fr		6 (26.1%)	10 (41.7%)		
**Hemodialysis filter**					
0.2 m^2^		6 (26.1%)	0	-	0.024
0.6 m^2^		11 (47.8%)	13 (54.2%)		
0.9 m^2^ or bigger		6 (26.1%)	8 (33.3%)		
oXiris®		0	3 (12.5%)		
**Localisation of the haemodialysis catheters**					
Internal jugular		15 (65.2%)	18 (75.0%)	-	0.188
Femoral		5 (21.7%)	6 (25.0%)		
Subclavian		3 (13.0%)	0		
**Initial settings for CRRT**					
Blood flow rate (mL/min)		56.5 (36–150)	75.0 (40–150)	-	0.177
Dialysate rate (mL/1.73m^2^/h)		2090 (825–8355)	2054 (1750–4550)	-	0.843
Replacement rate (mL/kg/h)		37.0 (29–61)	36.0 (28–53)	-	0.164
**Duration of CRRT (hours)**		34.0 (8–189)	45.0 (12–228)	-	0.286
**CRRT starting time**					
0–12. h		19 (82.6%)	22 (91.7%)	-	0.497
12–24. h		3 (13.0%)	2 (8.3%)		
>24. h		1 (4.3%)	0		
TPE sessions		-	3.0 (1–7)	-	-
ALT (IU/L)	0 h	34.0 (5–534)	48.0 (7–4388)	20 (4–1620)	<0.001
	24 h	44.0 (7–301)	37.0 (8–2626)	31.0 (2–922)	<0.001
	0–24 h change	+10.5 (−234, 101)	−10.0 (−1762, 23)	+12.0 (−698, 134)	<0.001
AST (IU/L)	0 h	68.0 (19–1070)	98 (19–8620)	35.5 (8–1954)	0.002
	24 h	61.5 (10–730)	89.0 (20–5747)	39.0 (1–1235)	<0.001
	0–24 h change	−8.5 (−343, 145)	−6.0 (−4523, 21)	2.0 (−765, 234)	0.003
HCO_3_ (mmol/L)	0 h	16.9 (9–26.5)	13.7 (9–35.3)	20.1 (3.5–37)	0.113
	24 h	23.1 (7–29.5)	23.9 (6.2–32.3)	22.8 (8.4–29.0)	0.351
	0–24 h change	5.0 (−10, 13)	6.0 (−8, 22)	3.0 (−11, 15)	0.773
Lactate (mmol/L)	0 h	4.5 (0.5–12)	3.0 (0.5–16)	2.25 (0.5–17)	0.005
	24 h	2.1 (0.9–11.6)	2.3 (1–9.7)	2.0 (0.1–14)	0.167
	0–24 h change	−2.5 (−9.15, 7.0)	0.5 (−6.7, 6.3)	−0.2 (−5.4, 9.2)	0.114
